# Identifying Individual T Cell Receptors of Optimal Avidity for Tumor Antigens

**DOI:** 10.3389/fimmu.2015.00582

**Published:** 2015-11-18

**Authors:** Michael Hebeisen, Mathilde Allard, Philippe O. Gannon, Julien Schmidt, Daniel E. Speiser, Nathalie Rufer

**Affiliations:** ^1^Department of Oncology, Lausanne University Hospital Center (CHUV), University of Lausanne, Epalinges, Switzerland; ^2^Ludwig Center for Cancer Research, University of Lausanne, Epalinges, Switzerland; ^3^TCMetrix Sàrl, Epalinges, Switzerland

**Keywords:** melanoma, immunotherapy, cytotoxic T cells, TCR affinity, TCR structural avidity, tumor antigens, T cell functionality, NTAmers

## Abstract

Cytotoxic T cells recognize, via their T cell receptors (TCRs), small antigenic peptides presented by the major histocompatibility complex (pMHC) on the surface of professional antigen-presenting cells and infected or malignant cells. The efficiency of T cell triggering critically depends on TCR binding to cognate pMHC, i.e., the TCR–pMHC structural avidity. The binding and kinetic attributes of this interaction are key parameters for protective T cell-mediated immunity, with stronger TCR–pMHC interactions conferring superior T cell activation and responsiveness than weaker ones. However, high-avidity TCRs are not always available, particularly among self/tumor antigen-specific T cells, most of which are eliminated by central and peripheral deletion mechanisms. Consequently, systematic assessment of T cell avidity can greatly help distinguishing protective from non-protective T cells. Here, we review novel strategies to assess TCR–pMHC interaction kinetics, enabling the identification of the functionally most-relevant T cells. We also discuss the significance of these technologies in determining which cells within a naturally occurring polyclonal tumor-specific T cell response would offer the best clinical benefit for use in adoptive therapies, with or without T cell engineering.

## What Defines a Protective CD8 T Cell Response?

### Antibody- and T Cell-Based Immunotherapies

During the last few years, immunotherapy has brought significant progress to clinical oncology. Major breakthroughs were made for melanoma patients (1–4), and progress becomes also evident for patients with frequent diseases, such as lung and kidney cancer (5). Specifically, immunotherapy aims at mobilizing the body’s immune cells to fight against cancer in highly specific ways. Several strategies have been developed over the last two decades to exploit the therapeutic potential of T cells (6). Administration of high-dose IL-2 (7) and tumor-associated (TA)-specific monoclonal antibodies (mAbs) (8) has initially provided long-term clinical benefits, albeit only for relatively few patients. More recently, mAbs that target immune checkpoints have shown remarkable results. In 2010, the successful outcomes of randomized phase III clinical trials with the anti-CTLA-4-specific mAb Ipilimumab offered strong clinical evidence that in humans, as in experimental animal models, the host’s immune system can control tumor growth (9). So far, several antibody-based drugs (anti-CTLA-4 mAb Ipilimumab-Yervoy, anti-PD-1 mAb Nivolumab-Opdivo, and anti-PD-1 mAb Pembrolizumab-Keytruda) have been approved for the treatment of melanoma, and the first FDA approval for carcinoma took place earlier this year, with the introduction of Opdivo for routine therapy of patients with non-small-cell lung cancer. Importantly, the rapidly increasing use of these antibodies represents a major breakthrough in the treatment of cancer patients (1, 2, 4, 9–11).

Already prior to immune checkpoint inhibitors, several lines of evidence suggested that antitumor immune responses might correlate with clinical outcome in patients with cancers. Among them is the very frequently observed correlation between the presence of tumor-infiltrating CD8 T cells and the improved clinical outcome for patients with solid tumors (12–14). The notion that antitumor T cells play a major role in controlling tumor growth was also largely demonstrated in clinical trials with adoptive transfer of autologous tumor-infiltrating T lymphocytes (TILs) (15). Even though technically and clinically challenging, the results are promising in terms of objective clinical responses and durability of responses (3, 16, 17). Moreover, genetic modification of T cells before adoptive cell transfer, such as inserting T cell receptors (TCRs) (18) and chimeric antigen receptors (19), was shown to further increase the clinical efficacy.

Despite that both antibody- and T cell-based immunotherapies can improve clinical outcome in cancer patients, multiple challenges still lay before us to improve the efficacy of cancer immunotherapies in the clinic. Indeed, many patients continue to experience disease relapse and/or progressive disease despite receiving these novel immune-based treatments. The potential reasons for this cancer “resistance” and “evasion” are based on cancer cell-internal and -external mechanisms, i.e., the two backbones of malignant diseases. For the former, cancer cells develop intrinsic therapy resistance. As for the latter, cancer cells corrupt the surrounding tissue microenvironment to support their own growth and suppress the anticancer immune responses. In short, the tumor cells and their microenvironment become a “wound that never heals” (20).

### CD8 T Cells Play a Central Role in Tumor-Specific Immune Responses

The recent immunotherapy successes in clinical oncology were build on the profound experience acquired over the years in hemato-oncology, as well as an increase in understanding of the roles of T cells in generating a potent and sustained antitumor immune response. After allogeneic hematopoietic stem cell transplantation, the graft-versus-leukemia effects assure long-term remission of patients with hematological malignancies. Importantly, T cells play a central role in graft-versus-leukemia by controlling tumor growth, progression, and recurrence. Similarly, T cells are also essential players in generating a protective and durable immune response against solid tumors. T cells can act against both cancer cell-internal and -external resistance mechanisms. First, they can specifically target and directly destroy cancer cells. Second, they can revert a tumor-promoting microenvironment into a tumor-hostile one, by changing the patient’s tumor biology toward a “healing wound.” Moreover, as therapeutic successes depend on broad and long-term protection, T cells are important players since, in contrary to pharmaceutical drugs alone, they can generate a therapeutic memory. Therefore, by furthering the development of novel T cell-based therapies against cancer, therapeutic pressure could be applied simultaneously against both malignancy backbones, and tumor escape would be minimized.

For successful immune defense, activated antigen-specific T cells must reach high frequencies, differentiate into numerous and powerful effector and memory cells, and exert multiple functions. To achieve this, T cells must first be primed following the specific recognition by the TCRs of antigenic peptides bound to self-major histocompatibility complex (MHC) molecules (referred to as pMHC thereafter) at the surface of antigen-presenting cells (APCs). Second, upon differentiation and expansion, T cells must migrate and localize to the tumor bed. An essential point is that the TCR–pMHC interaction should be sufficiently strong to enable the efficient recognition of tumor antigens (which are naturally presented at low levels) and to trigger potent tumor-specific T cell effector functions. Finally, robust memory T cells must be established, assuring long-term immune responses for durable disease control.

**Figure 1:**
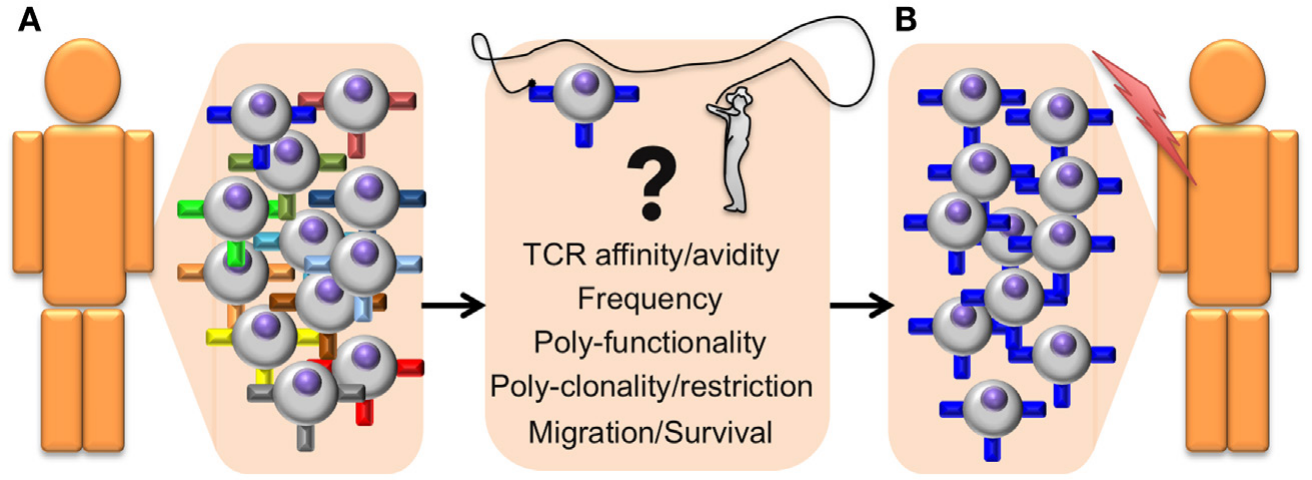
**Identifying antitumor T cells of high avidity and high function for adoptive cell transfer immunotherapy**. General outline presenting a step-by-step optimized protocol for the identification and adoptive transfer of the most potent tumor-specific CD8 T cells in cancer patients. **(A)** The selection of therapeutic autologous CD8 T cells (isolated from the tumor or from PBMC) is based on the following correlates of protection parameters that include structural TCR–pMHC affinity/avidity, T cell frequency, polyfunctionality (with differentiation and effector properties), poly-clonality, and poly-restriction to multiple antigens presented by different HLA alleles, cell migration capacity to the tumor site, as well as memory/survival properties with long-term persistence. **(B)** Selected T cells with optimal combination of those correlates will be isolated and expanded *ex vivo*, before being re-infused back in the patient. These selected tumor-specific CD8 T cell subpopulations should be highly effective at targeting and eliminating tumors *in vivo* and achieve enhanced and durable clinical benefits.

Since T cells play a major role in immune protection against cancer, it is important to determine which T cell properties are essential to achieve clinical benefit. Several “correlates of protection” have been identified; TCR–pMHC binding affinity/avidity, T cell frequency, poly-functionality, poly-clonality, poly-restriction (i.e., T cells specific for multiple antigens that are presented by different HLA alleles), migration to the tumor, and survival/persistence (21) (Figure 1). The assessment of these criteria can greatly help distinguishing between powerful and ineffective antitumor T cell responses and thus provides essential information on the quality of a patient’s immune response. In this review, we will specifically focus on T cell avidity, both in terms of TCR–pMHC binding properties and functional capacities.

### Functional Avidity of CD8 T Cells

The functional avidity is a biological measure that describes how well a T cell responds *in vitro* to a given peptide concentration. It is determined by the *in vitro* quantification of T cell functions, such as cytotoxic activity, IFN-γ production, and proliferation. Pioneering the field more than 20 years ago, we demonstrated that low T cell avidity is sufficient for *in vitro* proliferation or cytotoxicity to peptide-coated target cells but not for *in vivo* protection (22), a finding that was subsequently confirmed and extended by others (23). Meanwhile, there is a general consensus that CD8 T cell responses with increasing functional avidity are better in controlling virus infections (24, 25). There exists a close relationship between T cell functional avidity and target cell recognition, as shown in several antigenic systems (23, 24, 26–29).

In 1998, an elegant study revealed important insights into the roles of essential parameters for *in vivo* protection from lymphocytic choriomeningitis virus (LCMV) in mice (30). Besides the functional avidity of T cells, the authors analyzed the density of peptide antigen on infected cells, the binding strength of peptide to MHC, the magnitude of T cell responses, and the broadness of the TCR repertoire. They found that the T cells specific for the NP396 peptide provided the highest protection, based on their highest functional avidity and strongest binding of NP396 to MHC. Interestingly, the least protective were the T cells specific for GP33, despite their highest magnitude and TCR diversity, and a GP33 peptide density on infected cells about sixfold higher than of NP396. Finally, T cells specific for GP276 showed an intermediate potency for protection, based on intermediate functional avidity, but lowest TCR diversity, to the peptide with the lowest density (30). Also for T cell responses against tumors, results obtained from both mouse and human models suggest that T cells of high functional avidities (31) and strong peptide binding to MHC (32) are required for efficient protection.

Together, functional avidity stands out as a highly important correlate of protection (Figure 1). Nevertheless, functional avidity has yet to be evaluated much more systematically in the development and routine application of immunotherapy. Limitations of laboratory techniques are the major reasons why this is infrequently done. In general, T cell assessment is mostly limited to assays measuring antigen specificity, target cell killing and cytokine production (e.g., IFNγ) to fixed stimulation doses (17, 33–36). Importantly, these functional assays do not directly measure the TCR–pMHC affinity or avidity (Figure 1), despite representing a major determinant of T cell responsiveness and possibly a more relevant metric of the T cell response.

## What Defines an Optimal Self/Tumor Antigen-Specific CD8 T Cell Response?

### TCR–pMHC Affinity, Avidity, and Structural Avidity

**Figure 2:**
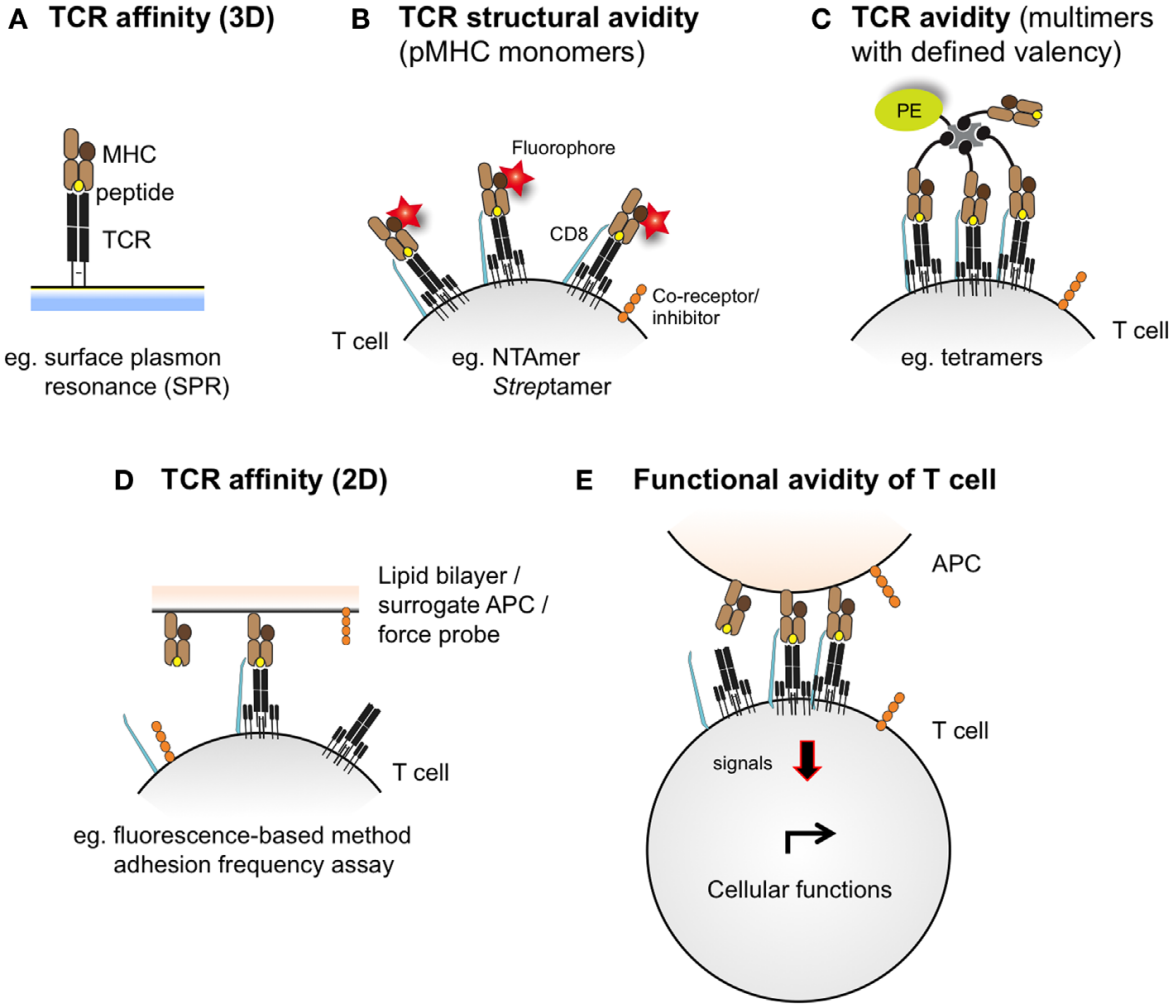
**Schematic representations integrating the different assessment levels of TCR–pMHC binding interactions**. TCR–pMHC affinity **(A)** refers to the binding strength of one TCR to one pMHC complex and is typically assessed by SPR (also defined as 3D interaction). At the cellular level (e.g., living antigen-specific CD8 T cells), the TCR–pMHC structural avidity **(B)** refers to the strength of interaction between monovalent TCR–pMHC complexes, as measured by reversible multimers (e.g., NTAmers, *Strep*tamers). Importantly, monomeric binding measurements contrast to the multimeric TCR–pMHC binding avidity **(C)**, which integrates the binding strength of multiple TCRs and pMHC complexes and is conventionally assessed by fluorescent pMHC multimers of known valency (e.g., tetramers). Recently, 2D-kinetic measurements **(D)** enable the assessment of TCR–pMHC binding affinity directly at the interface between a living T cell and a juxtaposed surface (e.g., a supported planar lipid bilayer or a surrogate APC) using fluorescent-based or micropipette adhesion frequency assays. T cell functional avidity **(E)** refers to the productive TCR–pMHC triggering integrating multiple TCR–pMHC binding interactions and represents the relative efficiency of T cell functionality as assessed in the presence of titrated peptide concentrations in various biological read-outs (e.g., target cell killing, cytokine production and proliferation potential).

T cell receptor–pMHC binding and kinetic interactions can be measured in terms of affinity or avidity. The TCR–pMHC binding affinity refers to the physical strength by which a single TCR binds to a single pMHC complex (37) and is inversely proportional to the dissociation equilibrium constant *K*_D_. Under equilibrium conditions, *K*_D_ is defined as the ratio of the dissociation rate and association rate (*k*_off_/*k*_on_), which are typically measured by surface plasmon resonance (SPR) (Figure 2). The rate at which the TCR dissociates from the pMHC complex, referred to as *t*_1/2_ (or half-life), represents another important parameter, and is related to the dissociation rate constant *k*_off_ by the equation *t*_1/2_ = ln 2/*k*_off_ (38). Conversely, the TCR avidity describes, in the cellular context, the association of multiple TCRs with their respective pMHC complexes (39). TCR–pMHC avidity depends on the TCR affinity and incorporates the potential effects of coreceptors (e.g., CD8), TCR density, and T cell functional (activation) status (40). Using the novel NTA–His tag-containing multimer technology (also termed NTAmers), we recently quantified monomeric TCR–pMHC dissociation rates (*k*_off_ or *t*_1/2_) of living tumor antigen-specific CD8 T cells (41, 42). Since NTAmers (TCMetrix, Epalinges, Switzerland) allow the quantitative assessment of TCR–pMHC binding interactions directly at the monomeric level and include the binding of the CD8 coreceptor, we now refer to this type of measurement as the structural TCR–pMHC avidity (42) (Figure 2). Finally, the overall CD8 T cell response is defined as the functional avidity and depends on the productive TCR–pMHC interactions, integrating the binding of multiple TCR–pMHC complexes and coreceptors together with the strength of cell–cell interactions (39).

### Antiself/Tumor-Specific CD8 T Cell Responses Are Mediated by TCRs of Low Affinity/Avidity

**Figure 3:**
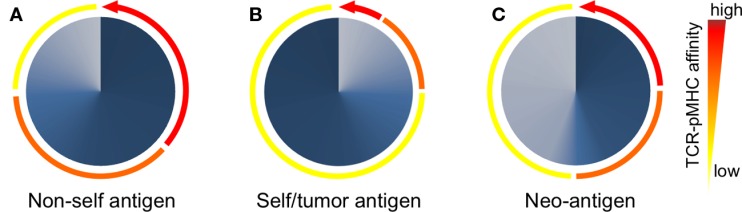
**Peripheral T cell repertoires available to respond to non/self- and self-antigens are shaped according to the TCR–pMHC affinities of individual T cells**. **(A)** After thymic selection, CD8 T cells specific for non-self (foreign) antigens express TCRs that span the entire physiological range from low (100 μM) to high (1 μM) affinity (depicted as colored arcs). In these non-self-specific repertoires, a large proportion (depicted as dark blue gradients) of T cells bear TCRs of intermediate to high-affinity TCRs (orange-red arcs). **(B)** Due to self-tolerance mechanisms, most but not all self/tumor antigen-specific T cells of high-affinity TCRs are deleted (red arcs). Consequently, T cell repertoires specific for self/tumor antigens are mainly composed (dark blue gradients) of low affinities (yellow arc). **(C)** T cells recognizing neoantigens are not deleted by self-tolerance mechanisms, since tumor-specific mutations generating neoantigens are “non-self like” epitopes. Thus, the repertoire of neoantigen-specific T cells is composed of increased proportions (dark blue gradients) of tumor-specific and high affinities TCRs (red arc).

The TCR–pMHC affinity and avidity vary dramatically between self and non-self-antigens (Figure 3). TCRs that interact with non-self peptides are frequently found among naive T cells and cover the whole physiological affinity range (29), with a preferential distribution of *K*_D_ found between 25 and 1 μM (43). In fact, it is now commonly accepted that immune responses to pathogens are dominated by cytotoxic T cells that express high-affinity TCRs (44, 45). By contrast, mature CD8 T cells specific for self/tumor antigens express TCRs of weak TCR–pMHC affinity (43), whereas high-affinity cells are very rare due to mechanisms of central and peripheral tolerance (46) (Figure 3). The *K*_D_ values of these interactions are typically in the range of 200–10 μM (with the mean around 100 μM) (43). Indeed, most TA antigens, such as cancer testis antigens (e.g., NY-ESO-1 and MAGEs) and differentiation antigens (e.g., Melan-A/MART-1, gp100, and tyrosinase) (47, 48), are expressed in the thymus (49). Consequently, thymocytes with high TCR–pMHC affinity/avidity for these antigens are negatively selected. Self/tumor-reactive T cells can further be eliminated in the periphery through mechanisms of peripheral tolerance (50). Nonetheless, these mechanisms spare cytotoxic T cells that can react to self/tumor antigens with relative low TCR–pMHC affinity/avidity (51–53) but which might be too low to mediate an effective antitumor immunity (Figure 3). Therefore, increasing the TCR–pMHC affinity and/or avidity of tumor-specific T cells is of particular interest for immunotherapy based on adoptive T cell transfer.

### Improving TCR–pMHC Affinity/Avidity Against Cancer Cells

Strategies developed to improve TCR–pMHC affinity involve the modification of TCR sequences by inserting point mutations within the complementary-determining regions (CDRs) of the variable domains of TCR αβ chains (54), and followed by the screening of yeast or phage variant libraries (55, 56). Such approaches result in the generation of TCR variants with supraphysiological binding strengths for peptide-MHC ligands up to the picomolar affinity range (55–57). However, as yeast and phage display technologies rely on large libraries and are based on random mutagenesis, the generation of such TCR mutants may be associated with loss of target cell specificity. In fact, T cells engineered with TCRs of very high affinities (*K*_D_ < 1 nM) become crossreactive (or alloreactive) to other pMHC complexes (58–60). To overcome this problem, TCR–pMHC affinity can be optimized in a highly controlled manner by structure-based methods (61–63). These approaches consist of *in silico* analyses made on available crystallographic structures of TCR–pMHC complexes and aim at identifying the key residues critically involved in the TCR–pMHC interactions. Such residues can subsequently be replaced with other structurally compatible ones, resulting in either increased or decreased TCR–pMHC affinity (64). The structure-based design of TCRs allows an increase of the TCR–pMHC affinity, while preserving antigen specificity and avoiding broad crossreactivity to other pMHCs (62, 65).

Through stepwise rational design of TCR–pMHC affinity (63, 64), we created a unique panel of human CD8 TCRs specific for the cancer testis antigen NY-ESO-1_157–165_ presented by HLA-A*0201 (defined as A2 thereafter) (66). This affinity-optimization process resulted from the introduction and combination of point mutations either within the CDR2α and CDR2β regions, and/or within the CDR3β region (63, 64). The TCR–pMHC binding and kinetic parameters (*K*_D_, *k*_off_, and *k*_on_) derived from SPR confirmed the *in silico* predicted changes in TCR affinity for A2/NY-ESO-1_157–165_, with *K*_D_ values ranging from 100 μM to 15 nM (65). By characterizing the functional potential of T cells expressing this panel of affinity-optimized TCRs, we found that T cells expressing TCRs with affinities lying in the upper physiological range (*K*_D_ from 5 to 1 μM) displayed greater biological responses (e.g., cell activation, signaling, proliferation, cytokine/chemokine secretion, and target cell killing) than T cells expressing intermediate/wild-type TCRs (*K*_D_ at 21.4 μM) or very low affinity (*K*_D_ > 100 μM) (65–67).

Strikingly, further increase within the supraphysiological TCR affinity range (*K*_D_ < 1 μM) led to drastic functional decline, with impairment in global gene expression and surface expression of activatory/costimulatory receptors (65, 67). Yet, such engineered T cells retained a high degree of antigen specificity toward the cognate pMHC (65). Importantly, T cell effectiveness was limited by at least two mechanisms. We observed preferential PD-1 expression on T cells expressing very high TCR affinities, as well as a full functional recovery upon PD-1 ligand blockade. In contrast, the upregulation of SHP-1 and SHP-2 phosphatases was broad, with the gradual enhanced expression in engineered T cells along the TCR affinity gradient (67). Together, these observations revealed that maximal biological activity, for the panel of human A2/NY-ESO-1_157–165_-specific engineered CD8 T cells, occurred within a well-defined affinity window with *K*_D_ ranging from 5 to 1 μM, above which T cell effectiveness was limited by TCR-mediated regulatory mechanisms (67).

### TCR–pMHC Affinity Threshold for Maximal Antitumoral CD8 T Cell Responses

Our findings nicely fit with other studies performed both in mice and human models, as well as in pathogen- and self/tumor-specific T cell responses strongly supporting the notion that T cell activation, signaling and subsequent function are limited to a given TCR–pMHC affinity window. It is known that a minimal threshold of TCR–pMHC binding is required for CD4 and CD8 T cell activation (68–72). Moreover, under physiological conditions, i.e., within the natural TCR affinity range (*K*_D_ from 200 to 1 μM) and under low peptide concentrations, numerous studies now provide strong evidence that enhanced TCR–pMHC affinity (*K*_D_) or off-rate (*k*_off_) correlates with improved T cell responsiveness (59, 60, 62, 65, 66, 73–83). However, the correlation between TCR–pMHC affinity and T cell function is not linear as there is a decrease in functionality for TCR–pMHC interactions taking place beyond the natural TCR affinity range (*K*_D_ < 1 μM) (65, 67, 81, 84, 85) or with prolonged half-lives (75, 77, 78, 80, 86).

Kalergis and colleagues were the first to propose that T cell activation might occur within an optimal range of half-life for TCR–pMHC interactions (78). Using altered peptide ligands derived from the ovalbumin OVA_257–264_ peptide, they showed that the duration of TCR–pMHC interactions (*t*_1/2_) regulated effector function and tumor-killing capacities of OT-1-specific CD8 T cells (80). Specifically, intermediate TCR–pMHC half-lives induced the strong expression of cytotoxic effector molecules, cytokine secretion, and consequently the efficient *in vivo* tumor clearance mediated by cytotoxic T lymphocytes (CTLs) (80). In line with these observations, Corse et al. studied the effect of TCR–pMHC interaction parameters on *in vivo* CD4 T cell activation, effector and memory responses upon immunization with lipopolysaccharide and moth cytochrome *c* (MCC) peptide, or related ligands exhibiting a range of TCR–pMHC half-lives (77). They found that the *in vivo* response to a superagonist ligand for the MCC-reactive TCR was associated with attenuated intracellular signaling, proliferation and effector functions over time following immunization. These data pointed to an upper limit of T cell ligand potency *in vivo*, with optimal T cell responses occurring to TCR–pMHC interactions of intermediate half-lives (77). Similar conclusions were reached in another study using vaccination with a panel of AH1 peptide ligands of increasing affinities specific for the immunodominant H-2Ld-restricted antigen from the colon tumor CT26 (84). Data from this report revealed that although TCR–pMHC affinity correlated well with the functional activity of the T cell clone *in vitro*, only vaccination with peptide ligands of intermediate affinities elicited functional T cell responses and provided best tumor growth control in BALB/c mice. Lastly, Kranz and colleagues explored the functional impact of increased TCR–pMHC affinity using the well-known 2C TCR-based mouse model (54, 55, 87). Excessive enhancement of TCR affinity through yeast display resulted in crossreactivity with other cognate pMHCs (58). Furthermore, they reported rapid *in vivo* disappearance of specific CD8 T cells expressing the m33 TCR of nanomolar affinity through mechanisms of peripheral deletion in both the TIL population and lymphoid organs, suggesting that TCR affinity controlled the survival and tumor infiltration of the transferred T cells (88, 89).

### Clinical Trials with TCR Affinity-Improved T Cells Against Tumor Antigens

The importance of TCR–pMHC binding parameters in the context of human antitumoral T cell mediated responses was originally demonstrated in clinical trials whereby melanoma patients received autologous peripheral blood mononuclear cells transduced with specific TCRs against the differentiation tumor antigen Melan-A^MART-1^_26–35_ (90). Compared to the native low affinity TCR isolated from the patient’s TIL, referred as DMF4 [*K*_D_ 29 μM (91)], with moderate ability to recognize the tumor antigen, Johnson and coworkers (92) showed a trend of improved clinical efficacy for the DMF5 TCR of higher affinity [*K*_D_ 5.6 μM; (91)]. Whereas objective responses were seen in 30% of patients who received the DMF5 TCR, several patients experienced in addition the destruction of normal melanocytes in the skin, ear, and eye, which led to uveitis and hearing loss (92). Similar studies based on the A2/NY-ESO-1_157–165_ tumor antigen model showed that specific CD8 T cells engineered to express a TCR (defined as 1G4) with affinity lying just beyond the natural affinity range (at *K*_D_ 730 nM) were those that displayed maximal functionality with the lowest crossreactivity *in vitro* (59). Importantly, clinical trials conducted with this affinity-improved TCR in adoptive cell transfer of autologous engineered T cells in patients with metastatic melanoma and sarcoma led to objective clinical responses without major adverse events (93). Recently, Tan et al. (81) examined the impact of TCR affinity on the functional properties of human transduced CD8 T cells of three different TA peptide antigens (hTERT_540–548_, NY-ESO-1_157–165_, and MageA3_161–169_) across a wide range of binding parameters. These authors found that the TCR affinity controls T cell antigen sensitivity and poly-functionality, further supporting the presence of an optimal range for CD8 T cell functional improvement but which varies with antigen specificity. Collectively, our data and those by others performed in the human tumor setting recapitulate the findings from mouse models, showing that maximal T cell functional responses occurred at intermediate TCR–pMHC binding strengths (Tables 1 and 2).

**Table 1:** **Engineered tumor-specific CD8 T cells with affinity-optimized TCR panels**.

Self-tumor/antigen-specific model	TCR/pMHC affinity/avidity assay	*In vitro* functional assay	*In vivo* functional assay	Correlation between TCR/pMHC affinity/avidity and T cell functionality	Reference
6 NY-ESO1-spec TCR mutants^a^	SPR (*K*_D_, *k*_on_, and *k*_off_)	IFNγ avidity and killing		✓ SPR-*K*_D_-^High^ T cells display higher functional avidity but also crossreactivity	(60)
4 NY-ESO1- and 6 Melan-A-spec TCR mutants^a^	SPR (*K*_D_, *k*_on_, and *k*_off_)	IFNγ avidity and killing		✓ SPR-*K*_D_-^High^ T cells display higher functional avidity but also crossreactivity	(59)
9 NY-ESO1-spec TCR mutants^a,b^	SPR (*K*_D_, *k*_on_, and *k*_off_) and multimers (MFI, on/off-rate)	Killing avidity, proliferation and TCR clustering		✓ SPR-*K*_D_/*k*_off_ and multimer off-rate correlate with functional avidity until a supraphysiological TCR affinity threshold	(66)
9 NY-ESO1-spec TCR mutants^a,b^	SPR (*K*_D_, *k*_on_, and *k*_off_) and multimers (off-rate)	Killing, Ca^2+^ flux, IFNγ avidity, TNFα, Il-2/4/8, CD107a and AICD		✓ SPR-*K*_D_/*k*_off_ and multimer off-rate correlate with functional avidity until a supraphysiological TCR affinity threshold^d^	(65)
9 NY-ESO1-spec TCR mutants^a,b^	SPR (*K*_D_, *k*_on_, and *k*_off_)	TCR/CD8 modulation, signaling and gene expression		✓ SPR-*K*_D_ correlates with cell functionality until a supraphysiological TCR affinity threshold^d^	(67)
7 gp100-spec natural TCRs^c^	SPR (*K*_D_) and multimers (MFI)	Killing, Ca^2+^ flux, IFN-γ and ERK phosphorylation	Tumor-size and autoimmunity	✓ SPR-*K*_D_ and multimer MFI correlate with cell functionality until a supraphysiological TCR affinity threshold	(83)
5 NY-ESO1-/6-MAGE-A3-spec TCR variants^a^	SPR (*K*_D_, *k*_on_, and *k*_off_)	CD107a, IFNγ, TNFα, and IL-2 avidity		✓ SPR-*K*_D_ correlates with poly-functionality until a supraphysiological TCR affinity threshold^d^	(81)
9 NY-ESO-1-spec TCR variants^b^	NTAmers (MFI, off-rate)	Ca^2+^ flux avidity		✓ NTAmer off-rate correlates with Ca^2+^ flux avidity until a supraphysiological TCR affinity threshold^d^	(42)

*^a^TCRs were transfected/transduced within human primary CD8 T cells*.

*^b^TCRs were transfected/transduced within human SUP-T1 cells*.

*^c^TCRs were transfected/transduced within mouse CD8^+/−^ splenocytes*.

*^d^Correlation between TCR–pMHC affinity and T cell functionality is not linear with a functional decline for TCR–pMHC interactions taking place beyond the physiological affinity range at *K*_D_ < 1 μM*.

**Table 2:** **Tumor-specific CD8 T cell clones identified by the altered ligand peptide approach**.

Self-tumor/antigen-specific model	TCR/pMHC affinity/avidity assay	*In vitro* functional assay	*In vivo* functional assay	Correlation between TCR/pMHC affinity/avidity and T cell functionality	Reference
Mouse GP70-spec CTL clone versus 7 mimotopes	SPR (*K*_D_) and multimers (MFI)	IFNγ and proliferation avidity	Tumor-free survival	✓ SPR-*K*_D_ and multimer MFI correlate with functional avidity until a supraphysiological TCR affinity threshold^a^	(84)
Mouse OT-1 T cells versus 6 mimotopes	SPR (*t*_1/2_)	Killing, IFNγ, IL-2, CD69, CD107a, granzyme B, and granule polarization avidity	Tumor size, survival and T cell tumor infiltration	✓ SPR-*t*_1/2_ correlates with functional avidity until a supraphysiological TCR affinity threshold^a^	(80)
Human NY-ESO1-spec CTL clone versus 2 mimotopes	SPR (*K*_D_)	IFNγ, MIP-1β, Ca^2+^ flux, granule polarization, and target conjugation-avidity		✓ SPR-*K*_D_ correlates with functional avidity, target-cell conjugation, granule polarization potency, and internal Ca^2+^ stores depletion intensity	(76)
Human NY-ESO1-spec CTL clone versus 17 mimotopes	SPR (*K*_D_, *k*_on_, and *k*_off_)	Killing- and IFNγ-avidity		✓ SPR-*K*_D_ and *k*_off_ correlate with functional avidity when *k*_on_ varies little	(73)
Human hTERT-spec CTL clone versus 7 mimotopes	SPR (*K*_D_, *k*_on_, and *k*_off_)	CD107a, IFNγ, TNFα, and IL-2 avidity		✓ SPR-*K*_D_ correlates with poly-functionality until a supraphysiological TCR affinity threshold^a^	(81)

*^a^Correlation between TCR–pMHC affinity and T cell functionality is not linear with a functional decline for TCR–pMHC interactions taking place beyond the physiological affinity range at *K*_D_ < 1 μM*.

### Increasing Antitumor TCR–pMHC Affinity/Avidity is Associated with Autoreactivity

T cell immunotherapy against cancer should ideally require T cells expressing affinity-improved TCRs, which efficiently control tumor growth without inducing on-target autoimmune reactivity against normal tissues expressing the same self-antigen. Yet, several reports have shown that in contrast to T cells of low avidity, high-avidity tumor-specific T cell responses were often associated with autoimmunity (92, 94–96). Recently, using seven human gp100_209–217_-specific TCRs isolated from melanoma patients and covering the physiological affinity range (1–100 μM), Zhong et al. (83) carefully evaluated the TCR affinity threshold defining the optimal balance between effective antitumoral activity and autoimmunity *in vivo*. Their results revealed that T cell antitumor activity and autoimmunity were closely coupled, whereby increasing TCR affinity/avidity correlated with improved tumor regression but was also associated with severe ocular autoimmunity in adoptively transduced A2-K^b^ mice. Together, these observations suggest that a relatively low-affinity threshold may be required for the immune system to avoid self-damage (*K*_D_ around 10 μM for A2/gp100_209–217_) (83). Intriguingly, another recent study reported that two TCR variants of increased binding interactions for the WT1 self/tumor antigen compared to the wild-type TCR were safe and did not mediate autoimmune tissue infiltration or damage when transduced into peripheral CD8 T cells and transferred *in vivo* (97). These findings are supported by a recent clinical trial showing that the usage of T cells transduced with a TCR of increased affinity for the cancer testis antigen A2/NY-ESO-1_157–165_ [1G4 α95:LY, *K*_D_ at 730 nM; (59)] did not lead to autoimmunity in melanoma and sarcoma patients (93). These apparently contradicting results could be explained by the differences in distribution and expression levels of self/tumor antigen expression in normal tissues and in tumors. For instance, NY-ESO-1 is expressed in very restricted germinal tissues (e.g., in testis cells) and WT1 antigen is expressed at low levels in normal self-tissues. This contrasts with other self/tumor antigens, such as Melan-A, gp100, and tyrosinase, that are widely found in melanocytes of the skin, eye, and ear, and whose expression has been shown to lead to melanoma/melanocyte-associated autoimmunity (92, 96, 98). Consequently, the choice of antigen specificity for adoptive cell transfer of affinity-improved T cells is of crucial importance.

Neoantigens, which result from gene mutations or aberrant expression in tumor cells and whose expression is uniquely found in tumor tissues, may represent ideal and safe targets for T cell therapy (99). Recently, using whole-exome sequencing combined with MHC-binding algorithms, Robbins et al. (100) identified mutated antigens expressed on autologous tumor cells that were recognized by three TIL lines from three melanoma patients. Importantly, these patients demonstrated regression of bulky metastatic lesions after adoptive transfer of autologous TILs, suggesting that neoantigens were able to generate strong immune responses in those patients. Tumor-derived neoantigens can trigger potent T cell immunity (101), probably because they are perceived as foreign by the immune system, allowing neoantigen-specific T cells to escape negative selection and express high-affinity/avidity TCRs (102). Thus, similar to non-self specificities, T cells recognizing neoantigen should theoretically express TCR–pMHC affinities spanning the entire physiological range with possibly a large proportion of high-affinity TCRs (Figure 3).

### Off-Target Toxicities in Clinical Trials using TCR Affinity-Improved T Cells

Another important parameter to be considered when using TCR affinity-improved T cells for adoptive-based therapies is that such cells also bear the risk of increased crossreactivity to structurally related self-peptides, resulting in off-target toxicities (103). Recently, two patients treated with TCRs engineered for enhanced affinity toward the cancer testis HLA-A1/MAGE-A3 tumor antigen developed off-target recognition of a similar but not identical peptide from the cardiac muscle-specific protein Titin (104), resulting in cardiogenic shock and death within a few days of T cell infusion (105). Another clinical trial based on the infusion of autologous anti-HLA-A2/MAGE-A3 TCR-engineered T cells further revealed unpredictable adverse effects with a possible crossreactivity to the MAGE-A12 self-antigen expressed in rare neurons, and leading to neurologic toxicities and death in two patients (106). These results highlight not only the functional potency of affinity-improved T cells toward tumor target cells but also the urgent need for improved preclinical systems to carefully assess on-target and off-target reactivity (e.g., *in silico* proteome screens and *in vitro* peptide specificity assays), to ensure the safety of self/tumor-specific TCR-engineered T cells in future clinical trials (99, 103, 107).

Collectively, we and others (108, 109) propose that the rational design of improved self/tumor-specific TCRs for adoptive T cell therapy may not need to be optimized beyond the natural TCR affinity range (*K*_D_ < 1 μM) to achieve optimal T cell function and avoiding possibly unpredictable risk of crossreactivity. Indeed, antigen-specific T cells may only naturally function within a well-defined narrow range of affinities under most conditions to ensure optimal responses against foreign pathogens and minimal responses against autoantigens (109). In that regard, new technological strategies are currently required allowing identifying and selecting for those naturally occurring but rare self/tumor antigen-specific T lymphocytes of the highest TCR affinity/avidity and functional capacities within the physiological TCR affinity range (Figure 3), as described below.

## How Can we Identify Self-Tumor/Antigen-Specific CD8 T Cells of High TCR–pMHC Structural Avidity?

### Molecular and Cellular TCR–pMHC Binding Measurements

Early TCR–pMHC binding analyses were typically performed by SPR, which allows for the simultaneous detection of molecular kinetics (*k*_on_ and *k*_off_) and affinity (equilibrium dissociation constant *K*_D_) of TCR–pMHC interactions in a single assay where one of the two molecules is attached to a sensor chip and the other one is flowing in soluble form (also defined as 3D interactions) (Figure 2) (110). Extensive studies revealed that natural human TCR–pMHC interactions were of relative weak affinities, with *K*_D_ ranging from 500 to 1 μM, rapid off-rate and slow on-rates (46, 111, 112). However, an inherent caveat of SPR analysis is that it requires the laborious and expensive production of soluble TCRs and ignores the contribution of the binding of the CD8 coreceptor and/or other molecules present in the vicinity of the TCR to the overall TCR–pMHC avidity.

Recently, novel technologies have emerged that enable the deduction of *k*_on_ and *k*_off_ kinetics directly at the interface between a living T cell and a surrogate APC, or between a T cell and a supported planar lipid bilayer (also defined as 2D interaction) (Figure 2) (113–115). Such 2D-kinetic analyses were shown to correlate with T cell activation, proliferation and cytokine secretion (79), and calcium signaling (116) in both CD8 and CD4 subsets (117). These studies also highlighted the cooperative role of CD8 coreceptor binding to the preexisting TCR–pMHC complex in a two-stage Lck-dependent manner (118, 119). Unexpectedly, 2D binding parameters are poorly correlating with 3D kinetic measurements, when compared side-by-side [reviewed in Ref. (120)]. Recent models of induced rebinding for TCR triggering, taking into account the TCR clustering effect and conformational changes occurring after initial pMHC encounter, now offer a reconciliation to these initial contradictory reports (121). Although 2D approaches allow for the measurements of TCR–pMHC binding parameters in a more physiological way than 3D SPR technology, both approaches should therefore be viewed as complementary. Importantly, 2D analyses require specialized equipment and time, precluding for the rapid and high-throughput screen of living antigen-specific T cells that could be useful for adoptive cell immunotherapy, and currently limiting their application to fundamental research (122, 123).

### TCR–pMHC Binding and Kinetic Measurements by Multimeric pMHC Molecules

To better understand the biophysical parameters regulating T cell activation, numerous studies of TCR–pMHC binding parameters were conducted using soluble pMHC of well-defined valencies directly on living cells (122, 124–126). Despite these advances in TCR staining, attempts to use soluble pMHC multimers to precisely determine the TCR affinity/avidity provided ambiguous results. While some reports showed a relationship between antitumor functional responses and the staining brightness (MFI) of multimeric pMHC attachment to the cell surface TCRs (Figure 2) (27, 83, 127, 128), others demonstrated a clear lack of correlation (129–131) (Table 3). Thus, the level of TCR–pMHC staining intensity (MFI) does not consistently correlate to the TCR–pMHC affinity/avidity, nor to the underlying T cell responsiveness.

**Table 3:** **Characterization of natural tumor-specific CD8 T cell clones and lines**.

Self-tumor/antigen-specific model	TCR/pMHC affinity/avidity assay	*In vitro* functional assay	*In vivo* functional assay	Correlation between TCR/pMHC affinity/avidity and T cell functionality	Reference
≈10 human gp100/Melan-A-spec CTL clones/lines^a^	Multimers (MFI)	Killing avidity		✓ Multimer-^High^ T cells display higher functional avidity	(128)
10 human MAGE-A10 spec CTL lines^a,b^	Multimers (MFI)	Killing avidity		✓ Multimer-^High^ T cells display higher functional avidity	(27)
Mouse gp100/Tyr-spec CTL lines^a,c^	Multimers (%)	Killing and IFNγ avidity	Tumor size	× No correlation between multimer-parameters and functional avidity	(129)
8 human MAG-A10/Melan-A/NY-ESO1 CTL clones^a,b^	Multimers (MFI, off-rate)	Killing avidity		✓ Multimer off-rate (but not MFI) correlates with functional avidity	(132)
Human NY-ESO1-spec CTL clones/lines^a,d^	Multimers (MFI)	Killing avidity		✓ Multimer MFI correlates with functional avidity	(127)
12 human Tyr-spec CTL clones^a,d^	Multimers (MFI/%, off-rate)	Killing, IFNγ, TNFα, Il-2/5/13, and GM-CSF avidity		× No correlation between multimer parameters and functional avidity	(131)
≈60 human NY-ESO1/Melan-A-spec CTL clones^b^	NTAmers (MFI, off-rate)	Killing avidity		✓ NTAmer off-rate correlates with functional avidity	(42)
≈100 human Melan-A-spec CTL clones^b^	NTAmers (MFI, off-rate)	Killing avidity and Ca^2+^ flux		✓ NTAmer off-rate correlates with functional avidity in CD8 T cell subsets	(41)

*^a^CTL clones/lines were derived through IVS*.

*^b^CTL clones/lines were derived from cancer patients*.

*^c^ADD-transgenic mouse model (gp100 and tyrosinase represent self-Ag)*.

*^d^CTL clones/lines were derived through allorestricted stimulation*.

Due to these discrepancies, multimeric association and dissociation kinetic rates from the T cell surface were evaluated as potential read-outs for determining TCR–pMHC affinity/avidity and correlates of T cell function. But again, several studies showed correlations between multimeric off-rates and antitumor T cell functions (62, 66, 132), whereas others failed to demonstrate any *in vitro* functional or *in vivo* protective association (82, 129, 131, 133) (Table 3). Major reasons for these inconsistencies include the kinetic bias generated by the multivalent nature of the pMHC complexes when compared to monomeric-based molecules, the impact of uncontrolled rebinding during dissociation assays (134), as well as by the induction of signaling events through TCR–pMHC multimerization that can trigger cell death (122, 134).

### Improved Detection and Isolation of Antigen-Specific CD8 T Cells by Reversible Multimers

Over the last decade, a major technological improvement was achieved with the development of reversible multimers (i.e., reagents in which pMHC monomers can be disrupted from the multimeric scaffold upon addition of a stimulus). By comparison with conventional multimers, reversible multimer staining allows for the isolation of practically “untouched” T cells, without inducing their TCR-mediated activation, phenotypic change, or activation-induced cell death (122). Knabel and colleagues (135) developed the first class of reversible multimers called *Strep*tamers (IBA, Goettingen, Germany). These molecules are made of fluorescent *Strep*Tactin, a derivative of streptavidin, coupled to several pMHC monomers carrying a streptag, a linear peptide optimized to bind to *Strep*Tactin (135). The molecule d-biotin binds *Step*Tactin with higher affinity than streptag and is able to compete for the same binding site, disrupting the multimeric complex. Consequently, the addition of free d-biotin releases the fluorescent *Strep*Tactin, breaking the multimers into non-activating pMHC monomers at the cell surface. Since MHC monomers do not stably bind to TCRs, they rapidly dissociate, allowing for the identification and isolation of antigen-specific CD8 T cells, while preserving their functional status, in contrast to conventional multimers (136).

Based on the same principle, pMHC monomers containing a desthiobiotin (DTB), a derivative of biotin that binds streptavidin with lower affinity, were multimerized using fluorescent streptavidin and used for sorting untouched antigen-specific CD8 T cells (137). Sorting and cloning of Melan-A-specific CD8 T cells using DTB-based multimers yielded over two times more clones than when using irreversible multimers, mainly because of avoidance of multimer induced apoptosis. Despite these technological advances, a major drawback of these reagents remained the lag time in the switch from multimeric to monomeric form and their weak molecular stability associated with their multimeric form, especially at high temperatures (122). More recently, Schmidt and colleagues (138) reported the third generation of reversible multimers, called NTAmers (TCMetrix), and made of His-tagged pMHC and fluorescent streptavidin carrying an engineered nitrilotriacetic acid (NTA) linker. NTAmer complexes are highly stable even at elevated temperature and have the advantage of decaying very rapidly into monomers, within 2–3 s upon addition of imidazole at doses that are non-toxic for T cells. Moreover, the benefit of using reversible NTAmers for sorting antigen-specific T cells without activation-induced cell death was further confirmed (138).

### Monomeric TCR–pMHC Off-Rate Measurements Specific for Non-Self Viral Antigens

Major efforts have been made to develop technologies enabling the measurements of monomeric TCR–pMHC binding strength and kinetics directly at the surface of live T cells. Indeed, rapid interrogation of these parameters may help discriminating CD8 T cells into high or low structural TCR–pMHC avidities (Figure 2) and thus permit the selection of T cells with optimal functional avidity (e.g., high T cell function and low autoreactivity). Thanks to the fluorescent labeling of individual pMHC monomers contained in the reversible multimeric complexes, it became possible to monitor and quantify monomeric dissociation of pMHC from the TCRs directly on living T cells. In 2013, Nauerth et al. (139) reported a real-time microscopic-based method using reversible two-color *Strep*tamers (IBA, Goettingen, Germany) to determine monomeric dissociation kinetics of non-self virus-specific TCRs on human and mouse T cells. The monomeric dissociation *k*_off_ rates were found to correlate with *in vitro* functional avidity as well as with *in vivo* protection capacity. CMV-specific CD8 T cells expressing TCRs with slow dissociation rates (long half-lives) were functionally superior to those with rapid dissociation rates (short half-lives) (139). The authors concluded that monomeric pMHC–TCR dissociation kinetics represent a valuable parameter to identify the best T cells for adoptive cell transfer, and to evaluate the quality of existing or induced immune T cell responses (140). However, the Achilles heel of the *Strep*tamer technology resides in the scaffold of the molecule itself. The significant lag time (about 60 s) required for the *Strep*tamer to decay into monomeric pMHC molecules after addition of free d-biotin as well as the photobleaching effect associated with the microscopic assay prevent for the precise determination of rapid TCR–pMHC off-rates, which are typically found within the self/tumor-specific CD8 T cell repertoires of lower TCR affinities. In turn, a great advantage is realized by the use of real-time fluorescence microscopy to enable simultaneous measurement of *k*_off_ on several CD8 T cells, typically as many as displayed by the field of view, and is as such not limited to clonal T cell populations.

### Monomeric TCR–pMHC Off-Rates Measurements Specific for Self/Tumor Antigens by NTAmers

**Figure 4:**
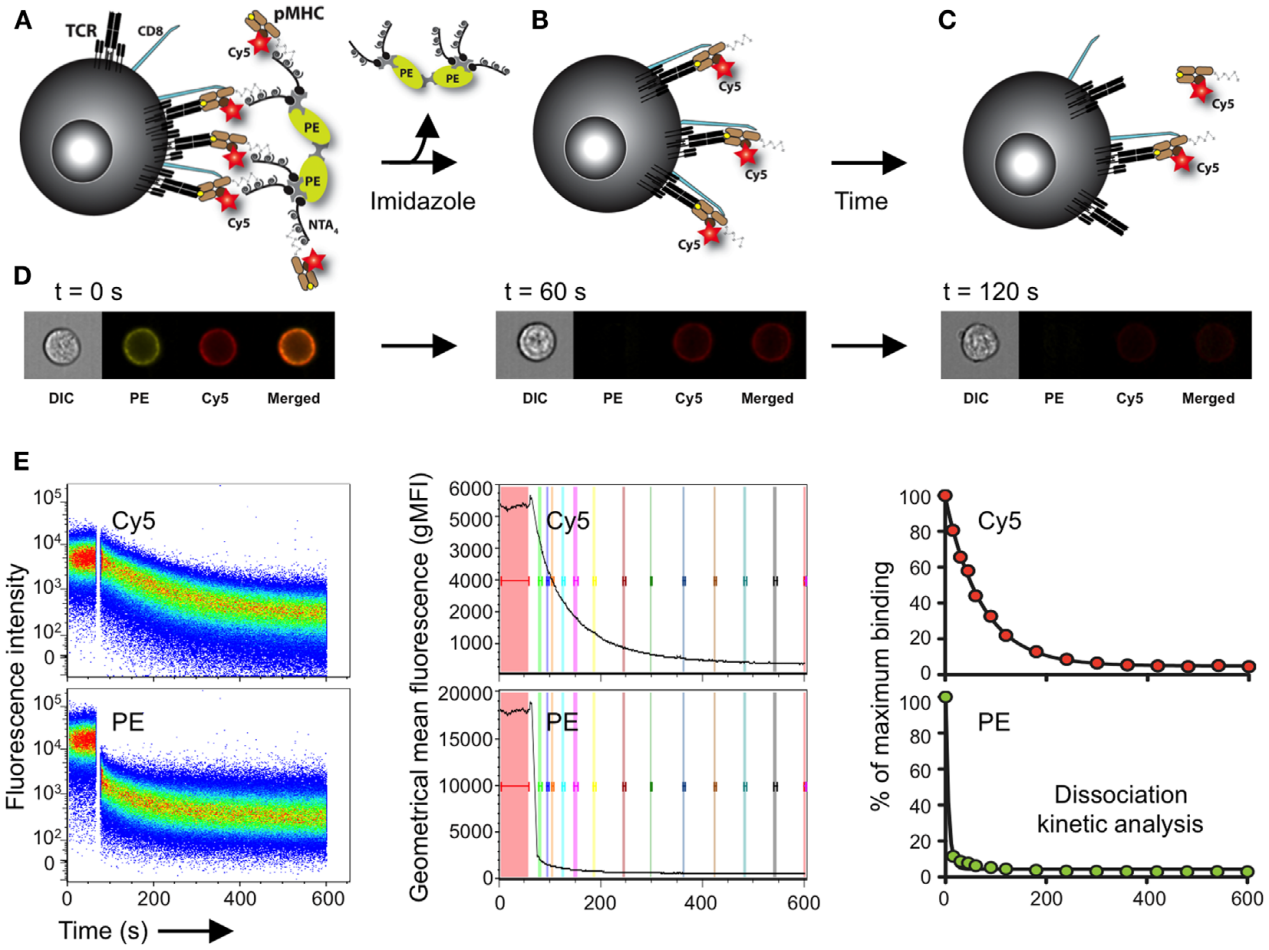
**Schematic representation of the NTAmer-based monomeric dissociation assay**. **(A)** CD8 T cells are stained at 4°C with multimeric NTAmers composed of streptavidin-PE (green)-NTA_4_ (gray) and peptide–MHC (brown) monomers containing His_6_-tag and Cy5-labeled β2m (red). **(B)** NTAmers are highly stable but upon addition of imidazole (100 mM), they rapidly decay in Cy5-labeled pMHC monomers and streptavidin-PE-NTA_4_ scaffolds. **(C)** Monomers subsequently dissociate from cell-associated TCRs (black) and CD8 (blue) according to the intrinsic TCR/CD8–pMHC dissociation rate (*k*_off_). **(D)** Representative DIC (differential interference contrast), PE, Cy5 and PE/Cy5 composite images acquired at the indicated time with a high-resolution microscopy flow cytometer (Amnis ImageStream^X^ Mark II) and illustrating the different stages **(A–C)** of the NTAmer dissociation assay. **(E)** Representative example of monomeric dissociation off-rates from a tumor-specific CD8 T cell clone following flow cytometry measurements by NTAmers. Imidazole is added after one minute baseline recording (left, white gap) and dissociation curves are followed over time within the Cy5 (monomers) and PE (NTA scaffold) channels. The kinetic module of FlowJo9 is used for geometric mean fluorescent intensity (gMFI) curve analysis (middle), while kinetic dissociation rates and half-lives are calculated with Prism (Graph Pad software Inc.). Adapted from Schmidt et al. (138) and Hebeisen et al. (42).

We recently used a two-color version of the reversible NTAmer molecule, in which pMHC monomers are made with Cy5-labeled β2m and complexed with PE-streptavidin (122) (Figure 4). The rapid decay (2–3 s) of the NTAmer complex into its pMHC monomeric constituents made it possible to precisely analyze by flow cytometry the dissociation kinetics of a wide spectrum of TCR–pMHC affinities, with a special emphasis for self/tumor-specific CD8 T cells (Figure 4). The reliability and accuracy of the NTAmer approach were validated by finding strong correlations between NTAmer-based monomeric dissociation rates and those obtained by SPR measurements (42), when applied on our previously described panel of TCR-engineered A2/NY-ESO-1_157–165_-specific T cells (65, 67). Using the NTAmer technology, we also successfully measured the monomeric TCR–pMHC dissociation rates of a large series of natural A2/NY-ESO-1_157–165_- and Melan-A^MART-1^_26–35_-specific CD8 T cell clones (*n* = 139) derived from various melanoma patients (42). Strikingly, the dissociation rates of tumor-specific CD8 T cells strongly correlated with their signaling and functional avidities, as determined by their capacity to mobilize calcium in response to TCR triggering and by their sensitivity to recognize and kill target cells (42) (Table 3). Thus, surface-based dissociation *k*_off_ enabled the discrimination of tumor-specific CD8 T cells of low and high functional avidities.

In summary, the NTAmer offers the real-time quantification of dissociation kinetics on a wide range of TCR–pMHC affinities directly at the surface of living, primary CD8 T cells, thus providing rapid, easy, and direct measurements of the monomeric TCR–pMHC dissociation rates within large numbers of tumor-specific CD8 T cell clones (41, 42) (Figure 4). Moreover, it is now possible to adequately evaluate the *ex vivo* impact of TCR–pMHC affinity/avidity on the functionality and differentiation of antitumor T cell responses in well-defined clinical settings. Since the NTAmer approach requires the cloning and expansion of antigen-specific CD8 T cells before measuring their TCR–pMHC dissociation off-rates, current efforts are devoted in translating the NTAmer technology to the single cell level.

### Impact of the CD8 Coreceptor on TCR–pMHC Affinity/Avidity

If accumulating evidence pinpoints the pivotal role of TCR–pMHC affinity/avidity in T cell activation capacity, several other molecular and cellular parameters were also shown to greatly impact on T cell responsiveness upon TCR triggering. As such, it is largely accepted that the αβ heterodimeric CD8 coreceptor enhances CD8 T cell activation via two main effects: (i) by recruiting p56^lck^ to the TCR/CD3 complex upon TCR engagement promoting cell signaling and (ii) by stabilizing the TCR–pMHC complexes through its weak but direct interaction with the α3 domain of pMHC class I molecule (40, 46, 118, 119, 141–143).

The biophysical contribution of CD8 attachment during TCR–pMHC triggering has been difficult to assess. Since the SPR technology does not allow the measurement of the dynamic effect of CD8 binding during TCR–pMHC interaction, its impact on TCR–pMHC kinetics were estimated at the surface of T cells with CD8-binding proficient or deficient pMHC tetramers (133, 143). Using a CD8 binding-deficient NTAmer variant, we recently performed precise measurements of the impact of CD8 attachment during monomeric TCR–pMHC binding dissociation assays (42). CD8 coattachment was found to strengthen the TCR–pMHC binding interaction by a factor of 3–4 times, as anticipated by previous tetramer dissociation assays (133, 143). Interestingly, the CD8 stabilization factor on TCR–pMHC dissociation was independent of the TCR–pMHC affinity, in contrast with the CD8 dependence for T cell activation, which can be directly linked to TCR affinity (65, 87, 133), and allows tuning the sensitivity and specificity of T cell responses (144).

Finally, the expression levels of various other receptors/molecules, as for instance costimulatory and inhibitory receptors, cytokine receptors, or adhesion molecules, can also be important for the modulation of T cell activation through the fine tuning of either TCR-dependent signaling pathways, T cell activation thresholds and/or T cell–APC adhesion-strength. Interestingly, while the expression level of these molecules can affect both the intensity and duration of T cell responses upon activation, we recently showed that some (e.g., PD-1 inhibitory receptor and SHP-1/SHP-2 phosphatases) also correlated with the TCR–pMHC affinity/avidity (67). Altogether, these additional receptors/molecules are part of a tunable system that enables T cells to adapt their reactivity to different stimulatory conditions. We have just begun to understand how those are achieved (108).

## Does the TCR–pMHC Structural Avidity Predict CD8 T Cell Functional Efficiency?

### Relationship Between TCR–pMHC Binding Parameters and CD8 T Cell Responsiveness

Up to date, a debate remains regarding which parameter(s) of the TCR–pMHC interaction (e.g., *k*_off_ and *K*_D_) could better predict T cell activation and function. While several reports showed that *k*_off_ was the most significant factor contributing to T cell activation (69, 78, 145–149), others reported that *K*_D_ was the preeminent correlate of T cell responsiveness (58, 74, 82, 84). Recent data suggested that these observations were in fact not in conflict which each other, but rather the manifestation of the association rate parameter (*k*_on_) (73, 150). Indeed, for TCRs with faster association rates to the pMHC (and thus high probability for TCR–pMHC rebinding), the affinity *K*_D_ is the better predictor of T cell activation potency. Conversely, for slower association rates (and low rebinding probability), the off-rate remains the better correlate (73, 150). Nonetheless, in most cases the *K*_D_ value is mainly driven by the *k*_off_.

Regardless of the type of parameter(s) used to quantify the TCR–pMHC binding interaction, data emanating from engineered TCR-variant panels (Table 1) or altered peptide ligand models (Table 2) have provided strong evidence, in the context of self/tumor-specific immune responses, that the functionality of CD8 T cells can be tailored by the TCR–pMHC affinity/avidity. As such, we and others demonstrate that within the range of physiological interactions (*K*_D_ 100–1 μM), the TCR–pMHC affinity (as determined by SPR) strongly correlates with various T cell functional read-outs (59, 60, 65–67, 73, 76, 80, 81, 83, 84). These include T cell potency for target cell conjugation, phosphorylation of downstream molecules of the TCR-signaling complex, intracellular Ca^2+^ mobilization, lytic-granule polarization, target cell killing, cytokine production, cell proliferation, polyfunctionality, *in vivo* tumor infiltration, and protection/survival.

### Identifying Rare, High-Avidity Self/Tumor-Specific CD8 T Cells in Melanoma Patients using the Novel NTAmer Technology

It is important to mention that most studies described above are based on artificial models (e.g., using affinity-optimized TCR variants) and therefore rely on a wide TCR–pMHC affinity spectrum (from *K*_D_ 100 μM to 1 pM) (Table 1). Since large-scale assessment of endogenous TCR–pMHC affinity/avidity repertoires has remained, until recently, technically challenging, only limited information is available on the overall impact and clinical relevance of TCR–pMHC affinity/avidity in the context of natural self/tumor antigen-specific CD8 T cell repertoires (Table 3). Specifically, the questions whether T cells of high TCR–pMHC affinity/avidity can be found in the endogenous tumor-specific repertoire of cancer patients and whether the TCR–pMHC structural avidity represents an important factor contributing to a robust antitumor T cell stimulation and activity remain open. To address them, novel tools had to be developed allowing the quantification of the endogenous TCR–pMHC affinity/avidity in relation to the tumor-specific T cell functions.

Recently, our group demonstrated that the NTAmer technology could be used to precisely assess the TCR–pMHC structural avidity on living TCR-engineered and natural self/tumor- specific CD8 T cells (42) (Figure 4). Using large panels of Melan-A^MART-1^_26–35_- and NYESO-1_157–165_-specific CTL clones isolated from vaccinated melanoma patients, we showed that the NTAmer-derived off-rates strongly correlated with the killing avidity of naturally occurring antitumor T cells (41, 42). Importantly, using this novel technology, we could quantify the potency of an immunotherapy intervention in melanoma patients. Indeed, we confirmed that the type of peptide used for vaccination of cancer patients profoundly influenced the TCR–pMHC structural avidity of tumor-specific T cells, which in turn correlated with T cell functions (41). Patients vaccinated repetitively with the natural Melan-A^MART-1^_26–35_ decapeptide generated tumor-specific CD8 T cells with increased TCR–pMHC structural avidities as compared to vaccinations with the analog Melan-A^MART-1^_26–35_ A27L peptide, even if the latter binds more strongly and stably to MHC as compared to the natural peptide. Analog peptides with enhanced MHC binding are commonly used for cancer vaccines. However, vaccination with enhanced MHC binding has likely similar consequences as vaccination with high peptide doses, since both result in the activation and selection of qualitatively inferior T cells, likely due to their lower functional avidity (23, 151). Indeed, we found that the overall functional properties of the tumor-specific CD8 T cells correlated with the biased T cell repertoire selection of vaccination with natural versus analog peptide (41, 152). Thus, vaccination with low peptide doses or peptides with weak/natural MHC binding favors an enrichment of T cell clonotypes with higher functional competence.

Consequently, the assessment of the TCR–pMHC structural avidity on living cells by NTAmers enabled to address which therapeutic vaccine protocol triggered the most potent, high-avidity tumor-specific T cell responses within comparative experimental cohorts, providing precious insights into the choice of peptide to be employed for future cancer vaccines. Furthermore, we were able to evaluate the impact of TCR–pMHC affinity/avidity on T cell differentiation. Our data reveal that, compared to a high-affinity mimotope vaccine, the use of the natural ­Melan-A^MART-1^_26–35_ peptide could impact both the functionality and the preferential differentiation of T cells bearing high structural avidity TCRs (41). Hence, we report the feasibility and usefulness of TCR–pMHC structural avidity assessment by NTAmers of naturally occurring polyclonal T cell responses, allowing the identification and selection of rare high-avidity cytotoxic T cells from patients for cancer therapy.

### A New Look at Old Questions

Several other hypotheses can now be re-evaluated to further dissect the impact of TCR–pMHC affinity/avidity on T cell (poly)functionality, memory formation, survival and persistence. In terms of functionality, additional studies need to precisely appraise the impact of TCR–pMHC affinity/avidity on the ensuing intracellular signals and effector activities, this in terms of both quantitative (ligand potency versus maximal activity) and qualitative (poly-functionality) aspects. Our current data indicate that the TCR–pMHC structural avidity correlates better with ligand potency (EC_50_) than with maximal activity using various functional readouts upon stimulation with titrated amounts of peptide [(41) own unpublished observations]. Of note, distinct T cell functions are triggered with different activation thresholds (Ca^2+^ flux < killing < cytokine-release < proliferation) (153–159), and could therefore be differentially affected by the TCR–pMHC affinity/avidity. Besides, more detailed studies are required to fully characterize the impact of TCR–pMHC affinity/avidity on the ability of CD8 T cells to develop distinct cytokine profiles (e.g., T_H_1 versus T_H_2) (131) or polyfunctional responses, a well-established and important indicator of the ability of CD8 T cells to control viral infections, as suggested in various models (160).

Another important question is whether there is a distinct regulation of the repertoire selection, differentiation and persistence of high- versus low-TCR–pMHC affinity/avidity antitumor CD8 T cells *in vivo*. For instance, numerous studies conducted in mouse models (70, 161–165) or *ex vivo* analysis of human T cell responses (166, 167) targeting pathogen-derived antigens demonstrate that the secondary repertoire is selectively enriched in high-affinity/avidity TCR-expressing T cells compared to the primary one. Such process of narrowing of the memory repertoire largely results from the loss of low-affinity/avidity TCR-bearing T cells during antigen-driven clonal expansion. Indeed, due to interclonal competition and restricted access to pathogen epitopes, low-avidity T cells are less likely to be primed and rapidly expanded than high-avidity ones (168–170). However, high-affinity/avidity immunodominant clonotypes may also be preferentially driven toward functional impairment, when compared to low-affinity/avidity ones, as a physiological consequence of T cell activation (171, 172).

T cell receptor–pMHC affinity/avidity could also represent a critical determinant for T cell susceptibility to tumor homing, as suggested in transgenic mouse models of adoptive transfer of high versus low-avidity tumor-specific CD8 T cells (173–177). When transferred into tumor-bearing mice, high-avidity CTLs were shown to be more potent than low avidity CTLs to infiltrate tumors (174, 175, 177), which could be partly attributed to the expression of integrins and lectins, such as CD62L and CD11a, on high avidity CTLs (174), and the recognition of tumor antigens (32), emphasizing the role of antigen as homing molecule. High-avidity tumor-infiltrating T lymphocytes (TILs) expressed higher levels of molecules linked with their killing potential (e.g., granzyme B and perforin), associated with reduced levels of inhibitory proteins (e.g., LAG-3, PD-1, and NKG2A), than low avidity TILs (174). In contrast, low-avidity T cells were shown to upregulate members of the apoptosis pathway (e.g., Bim, FasL, and CD24) promoting their own cell death but also that of other tumor-specific T cells in the tumor microenvironment (173, 174). However, high-avidity CTLs were also more prone to tolerization mechanisms, and were preferentially tolerized by dendritic cells or regulatory T cells in the tumor microenvironment, while low-avidity CTLs retained their effector functions (175–177).

## Conclusive Remarks

Immunotherapy of cancer has made significant progress with the recent introduction of new therapeutic reagents, such as antibodies specific for CTLA, PD-1, and PDL-1, so-called immune “checkpoints.” Yet, we still need robust techniques allowing the rapid identification and isolation of CD8 T cells of optimal avidity and functions against tumors. Ideally, these technologies should enable the efficient screening of live CD8 T cells derived from a tumor sample or the peripheral blood of cancer patients at the single cell level. In that regard, binding strength analysis with novel fluorescent pMHC systems (e.g., NTAmers and *Strep*tamers), combined with single cell microscopic or LCD camera screening may enable to retrieve cells with the desired dissociation rates, which could then be used for T cell amplification and/or TCR cloning. Among these selected clones, the ones that express TCRs of highest TCR–pMHC structural avidity could then be tested for other parameters that may define optimal antitumoral activity *in vivo*, including their differentiation status (e.g., naive, effector, and memory), surface activatory/inhibitory receptor profile (e.g., CD69, PD-1, and CTLA4), effector properties (e.g., target cell killing, cytokine and chemokine secretion, and poly-functionality), as well as their proliferation potential. The combination of these parameters should allow the rational selection for tumor-specific, high-avidity cytotoxic T cells that have maximal capacity to control tumor growth and eliminate tumor cells *in vivo* (Figure 1).

Furthermore, by developing NTAmers or related technologies for the quantification of TCR–pMHC affinity/avidity within polyclonal populations, it may become possible to characterize the impact of immune-based therapies (adoptive cell transfer and checkpoint blockade), as well as chemotherapy and radiotherapy, on the survival and persistence of anticancer T cells. The addition of the TCR–pMHC affinity/avidity read-out may offer a new “biometric,” by which the quality of the T cell response can be directly evaluated and graded in order to better characterize their impact on the efficacy of cancer-based therapies.

Finally, neoantigen-specific T cells are expected to express TCRs of higher affinity/avidity and to be safe to use to treat cancer patients (99), as T cells with such TCRs are not clonally deleted, are truly tumor specific and thus are unlikely to attack healthy cells (102). In the near future, more neoantigens will be identified (102), allowing for the isolation of neoantigen-specific T cells of optimal avidities using reversible multimer-based technologies. Neoantigen-specific TCR cloning may also give rise to libraries of potent transgenic T cells that can be used against common neoantigens shared by various cancers (e.g., BCR-ABL).

Understanding the correlates of immune protection and developing technologies and algorithms allowing selecting for the best (i.e., high avidity and high function) tumor-specific CD8 T cells will support the progress of T cell-based therapies against cancer.

## Author Contributions

MH, MA, PG, JS, DS, and NR wrote the review.

## Conflict of Interest Statement

The authors declare that the research was conducted in the absence of any commercial or financial relationships that could be construed as a potential conflict of interest.
